# Vascularites cutanées leucocytoclasiques: à propos de 85 cas

**DOI:** 10.11604/pamj.2017.26.138.9721

**Published:** 2017-03-14

**Authors:** Amina Aounallah, Aicha Arouss, Najet Ghariani, Wafa Saidi, Badreddine Sriha, Mohamed Denguezli, Colandane Belajouza, Rafia Nouira

**Affiliations:** 1 Université du Centre, Tunisie; 2 Service de Dermatologie, CHU Farhat Hached, Sousse, Tunisie; 3 Laboratoire d’Anatomie et de Cytologie Pathologique, CHU Farhat Hached, Sousse, Tunisie

**Keywords:** Vascularite cutanée leucocytoclasique, étiologies, évolution, Cutaneous leukocytoclastic vasculitis, causes, evolution

## Abstract

La présentation clinique, les étiologies et l'évolution des Vascularites leucocytoclasiques sont peu étudiées. L'objectif de notre travail est d'étudier les particularités épidémiologiques, cliniques, étiologiques et évolutives de cette entité. Nous avons mené une étude transversale portant sur 85 patients ayant une vascularite leucocytoclasique colligés dans le service de dermatologie de l'hôpital Farhat Hached de Sousse entre Janvier 2000 et Décembre 2013. Pour chaque patient, une fiche des données épidémiologiques, cliniques, paracliniques et étiologiques a été remplie. L'âge moyen de nos patients était de 47,65 ans avec des extrêmes allant de 10 à 78 ans. Cinquante trois femmes et 32 hommes étaient recensés (sexe ratio = 0,6). Les manifestations cutanées étaient dominées par le purpura vasculaire (88,2%). Les étiologies les plus fréquentes étaient les maladies systémiques (51%), les infections (20%) et les dermatoses neutrophiliques (14,5%). Les autres étiologies retrouvées étaient : les causes médicamenteuses (9,1%) et les hémopathies malignes (5,4%). Chez 30 patients (35,3%), l'étiologie n'a pas été retrouvée. Deux facteurs prédictifs à l'évolution aigue étaient retenus : la présence d'une infection récente (p= 0,014) et d'une prise médicamenteuse précédant l'éruption cutanée (p= 0,013). L'évolution chronique était corrélée positivement aux anticorps antinucléaires (p= 0,009), et à la cryoglobulinémie (p=0,025). Notre étude illustre la multitude des étiologies de vascularites leucocytoclasiques. La recherche d'une maladie sous-jacente est impérative afin de garantir une meilleure prise en charge thérapeutique.

## Abstract (en)

Clinical manifestation, etiology and outcome of leukocytoclastic vasculitis are little studied. The aim of our study was to examine epidemiological, clinical etiological, and evolutionary characteristics of this entity. We conducted a cross-sectional data collection from medical records of 85 patients with leukocytoclastic vasculitis in the Department of Dermatology at the Farhat Hached University Hospital, Sousse between January 2000 and December 2013. Epidemiological, clinical, paraclinical, etiological data sheets had been completed for each patient. The average age of patients was 47.65 years, ranging between 10 and 78 years. Fifty-three women and 32 men were registered (sex ratio = 0.6). Cutaneous manifestations were dominated by vascular purpura (88.2%). The most common causes of leukocytoclastic vasculitis were systemic diseases (51%), infection (20%) and neutrophilic dermatoses (14.5%). Other causes were drugs (9.1%) and hematologic malignancies (5.4%). The cause of leukocytoclastic vasculitis was not detected in 30 patients (35, 3%). Two predictive factors associated with the acute outcome were retained: the presence of a recent infection (p= 0.014) and drug intake before the rash (p= 0.013). Chronic evolution was positively correlated with antinuclear antibodies (p= 0.009) and cryoglobulinemia (p=0.025). Our study highlights the multitude of causes of leukocytoclastic vasculitis. The search for an underlying disease is an imperative in order to ensure better therapeutic management.

## Introduction

La vascularite leucocytoclasique (VLC) est la forme de vascularite la plus communément rencontrée en pratique dermatologique [1]. L'aspect clinique le plus fréquemment observé est le purpura vasculaire qui est caractérisé par un aspect infiltré, palpable avec une prédominance aux membres inférieurs. La vascularite leucocytoclasique est associée à un large spectre d'affections systémiques, de néoplasies, d'infections ou à une hypersensibilité médicamenteuse [1]. Plusieurs publications se sont intéressées aux vascularites leucocytoclasiques comme celles faites en Australie par Tai [2] ou au Kuwait par Al-Mutairi [3]. En Tunisie, à notre connaissance, aucune étude sur la VLC n'a été entreprise. L'objectif de notre travail était de déterminer le profil épidémio-clinique, paraclinique et évolutif des VLC, ainsi que les différentes étiologies associées.

## Méthodes

Il s'agissait d'une étude transversale dont le recueil des données a été fait rétrospectivement incluant tous les patients ayant été hospitalisés pour vascularite leucocytoclasique, au service de dermatologie de l'hôpital Farhat Hached de Sousse sur une période s'étendant du 1er janvier 2000 au 31 décembre 2013. Le critère d'inclusion principal était un examen anatomopathologique d'une biopsie cutanée concluant à une VLC. Le diagnostic de la VLC était posé devant la présence d'un infiltrat cellulaire fait de polynucléaires à noyau picnotique autour et dans la paroi des vaisseaux, associé à une nécrose fibrinoide. Pour chaque patient, nous avons recueilli les données épidémiologiques, les caractéristiques cliniques et paracliniques de la VLC au moment du diagnostic, les modalités thérapeutiques, l'évolution de l'affection et le bilan étiologique de la VLC.

## Résultats

**Figure 1:**
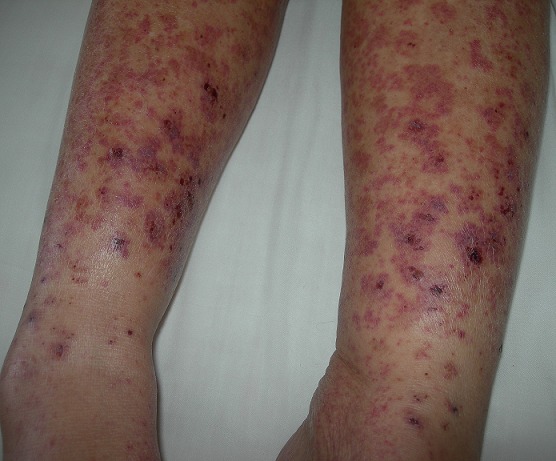
Vascularite purpurique des jambes

Quatre-vingt cinq patients suivis pour VLC ont été retenus sur une période d'étude s'étalant sur 14 ans (6 cas/an). Ils étaient répartis en 53 patientes de sexe féminin et 32 patients de sexe masculin avec un sex-ratio homme/femme de 0,6. L'âge moyen de nos patients était de 47,65 ans avec des extrêmes variant de 10 à 78 ans. La médiane d'âge était de 48 ans. Un pic de fréquence était noté dans la tranche d'âge comprise entre 40 et 49 ans (27,1%). Des antécédents personnels ont été notés chez 63,5% des patients (54 cas). L'hypertension artérielle (HTA) et le diabète étaient associés aux vascularites leucocytoclasiques cutanées dans 17,6% des cas pour le premier et 16,4% pour le deuxième. La durée moyenne d'évolution des symptômes était de 68,34 jours. La médiane était de 30 jours avec des extrêmes variant de 4 jours à 2 ans. Une évolution aigue (inférieure à 3 mois) a été notée chez 82,4% des patients (70 patients) alors qu'une évolution chronique (supérieure à 3 mois ou des épisodes récurrents) était notée chez 15 malades seulement (17,6% des patients). Dans notre série, un facteur déclenchant a été retrouvé chez 40 patients (47,1%) dont un épisode infectieux précédant l'éruption cutanée chez 29 patients (34,1%), et une prise médicamenteuse chez 32 patients soit (37,1%). Au moment du diagnostic, tous nos patients avaient présenté une atteinte dermatologique dont un purpura (Figure 1) dans 88,2% des cas. Il était pétéchial chez 27 cas (31,7%), ecchymotique chez 26 cas (30,6%), nécrotique chez 16 cas (18,8%) et bulleux chez 6 patients (7,1%). Nous retrouvions par ailleurs comme autres signes cutanés des ulcères dans 22,4% des cas, un livedo, une urticaire et des pseudo-folliculites dans 3,5% des cas respectivement et des nodules sous-cutanés dans 1,1% des cas. Les lésions étaient retrouvées exclusivement aux membres inférieurs chez 62,4% des cas, généralisées et diffuses à tout le tégument chez 30,6% des cas, et aux membres supérieurs ou inférieurs uniquement dans 7,1% des cas. Une atteinte extra-cutanée était retrouvée chez 43 patients (48,8%) à type d'arthralgies dans 31,8% des cas, d'atteinte digestive dans 15,3% des cas, d'atteinte ophtalmologique dans 13% des cas, d'atteinte oto-rhino-laryngologique dans 10,6% des cas et de neuropathie périphérique dans 5,6% des cas. Sur le plan biologique, un syndrome inflammatoire biologique était retrouvé dans 75,4% des cas. La C-réactive protéine et la Vitesse de Sédimentation avaient une valeur moyenne respectivement de 57mg/l et 48,68 s. Une insuffisance rénale organique était notée chez 4 cas (4,7%). Une protéinurie positive était détectée chez 3 cas (3,4%). Le bilan étiologique avait conclu à une vascularite cutanée secondaire chez 64,7% des cas et à une VLC idiopathique chez 35,3% des cas. Parmi les VLC secondaires aux maladies systémiques (51%), nous retrouvions 10 cas de purpura rhumatoide, six maladies de Behcet, trois syndromes de Gougerot Sjogren, deux cas de lupus érythémateux systémique, deux cas de cryogloblinémie mixte essentielle, deux cas de périartérite noueuse, un cas de vascularite urticarienne hypocomplémentémique, un cas de maladie de crohn et un cas de syndrome des anticorps antiphospholipides. En ce qui concerne les VLC associées aux dermatoses neutrophiliques, nous retrouvions 7 cas de syndrome de Sweet et 1 cas de pyoderma gangrenosum. Les VLC d'origine infectieuse étaient retrouvées dans 20% des cas. Les causes infectieuses étaient représentées par l'infection urinaire (n=3), la pyodermite (n=3), la primo-infection tuberculeuse (n=1), la gastroentérite parasitaire (n=2), les viroses (n=2). Les VLC médicamenteuses étaient retrouvées dans 9% des cas. Les différents médicaments responsables de VLC chez nos patients étaient l'acide méfénamique, l'ampicilline, la gabapentine, la metformine et l'amoxicilline dans respectivement 1 cas chacun. Les VLC avaient révélé trois cas d'hémopathies malignes : 2 cas de myélome multiple et un cas de leucémie à tricholeucocytes. Toutes les causes de VLC secondaires sont répertoriées dans le Tableau 1. Le repos au lit était préconisé chez tous nos patients en association à une corticothérapie générale (n=22), à la colchicine (n=30), et à d'autres thérapeutiques en fonction du terrain et de la pathologie associée (anti-inflammatoires non stéroïdiens, antibiotiques). Chez nos patients, deux facteurs étaient liés à une évolution aigue (<3mois) : l'existence d'une infection aigue précédant la vascularite leucocyclasique (p=0,013) ou d'une prise médicamenteuse connue (p=0,014) et deux facteurs étaient liés à une évolution chronique notamment la positivité des anticorps antinucléaires (p=0,01) et la cryoglobulinémie (p=0,025).

**Tableau 1:** Différentes étiologies des vascularites leucocytoclasiques cutanées secondaires chez nos patients

Etiologies secondaires	%	Nombre (N= 55)
Maladies de système	51	28
Infection	20	11
Dermatoses neutrophiliques	14,5	8
Médicaments	9,1	5
Néoplasies	5,4	3

## Discussion

La VLC est une inflammation des vaisseaux de petit calibre, caractérisée par un infiltrat inflammatoire associé à une leucocytoclasie (fragmentation des polynucléaires neutrophiles) et une nécrose fibrinoide au niveau des veinules post capillaires des petits vaisseaux [4]. Elle constitue le type histologique le plus fréquent des vascularites cutanées [1]. La VLC se manifeste souvent cliniquement par un purpura palpable siégeant sur n'importe quelle partie du corps mais volontiers déclive aux membres inférieurs [1]. L'incidence des VLC est d'environ 30 cas par million de personnes par an [2]. La VLC constitue 67,3% des vascularites cutanées dans l'étude de Leelavathi qui a porté sur 85 cas [5]. L'incidence de la VLC dans notre pays n'est pas connue faute de travaux à grande échelle. Dans notre étude nous avons recensé 85 patients ayant une VLC qui ont été hospitalisés au service de dermatologie de l'hôpital Farhat Hached de Sousse durant une période de 14 ans (6 cas/an). Cependant, ces chiffres sous-estiment certainement leur fréquence réelle puisque nous n'avons inclus que les patients hospitalisés sans tenir compte des malades non hospitalisés ou qui avaient consulté dans d'autres services. La VLC peut survenir à tout âge avec une prédominance de l'âge adulte [1]. En se référant aux données de la littérature, l'âge moyen des patients varie de 42,5 ans à 60 ans [2, 3, 5]. L'âge moyen de nos patients était de 47 ans. Selon les publications, le sexe ratio hommes/femmes au cours de la VLC varie de 0,7 à 1,8 [2, 3, 5]. Dans notre étude, il existe une prédominance féminine avec un sex-ratio hommes/femmes de 0,6. Les manifestations cutanées de la VLC sont diverses. Les signes cutanés peuvent être à type de purpura, d'urticaire, d'ulcères, de papules infiltrées purpuriques, de livedo réticulaire, de nodules et de gangrènes digitales [1, 3, 5]. Les lésions maculopapuleuses purpuriques sont les manifestations cutanées les plus fréquentes des vascularites retrouvées chez 76 à 100% des malades selon les séries [2, 3, 5]. Le purpura était aussi identifié comme le signe clinique le plus sensible au diagnostic de la VLC [3]. Il peut avoir un aspect pétéchial, ulcéré et/ou nécrotique [2, 3]. Dans notre étude, le purpura vasculaire était également le symptôme le plus fréquent, présent au moment du diagnostic chez 88,2% des patients. Il était à type de pétéchies dans 31,7% des cas. Le purpura siège volontiers aux membres inférieurs, favorisé par l'orthostatisme mais pouvant également siéger sur les cuisses, les fesses ou parfois de façon plus diffuse [1, 2]. Dans notre série, la topographie des lésions était similaire à celle rapportée dans la littérature. En ce qui concerne les manifestations extra-cutanées, elles se voient au cours de la VLC dans 20% à 50% selon les séries [2, 3, 6, 7]. Tous les organes peuvent être atteints [5]. L'atteinte systémique était retrouvée chez nos patients dans 48,8% des cas. Comme chez nos patients, les arthralgies constituent la manifestation extra-cutanée la plus fréquente variant de 8,3 à 50,8% selon les séries [2, 3]. Les autres manifestations extra-cutanées sont dominées par les manifestations rénales et digestives. Leur fréquence est variable selon les séries allant de 2 à 14% [2, 3]. Le diagnostic positif de la VLC est basé sur la biopsie cutanée. Celle-ci doit être pratiquée dans les 48 premières heures après l'apparition de la lésion pour être contributive [7]. Elle doit être suffisamment profonde pour pouvoir examiner l'hypoderme [1, 7]. Le diagnostic histologique est porté sur l'existence de dépôts fibrinoides au niveau des capillaires et/ou des veinules post-capillaires, de cellules inflammatoires en particulier des polynucléaires neutrophiles qui pénètrent la paroi du vaisseau, et d'une poussière nucléaire périvasculaire : la leucocytoclasie [7].

L'immunoflurescence directe a un intérêt fondamental dans le diagnostic étiologique des VLC, elle est positive dans 70,5% à 100% des cas [3, 8, 9]. Selon certains auteurs, lors de l'évaluation de la VLC à l'immunoflurescence directe, on trouve un dépôt d'IgA dans 35 à 88% des cas [3, 9]. Selon d'autres séries, l'immunoflurescence directe montre le plus souvent un dépôt de C3 (71%), suivie d'IgM (35%), d'IgA (12%) et d'IgG (8%) [7]. Sur le plan étiologique, le dépôt d'IgM suggère une maladie associée au facteur rhumatoïde telles que les cryoglobulinémies et lymphoproliférations monoclonales [7, 8]. Un dépôt prédominant d'IgA au niveau vasculaire est sensible pour le diagnostic de purpura rhumatoïde selon la majorité des publications [7, 8]. Dans notre série, l'immunoflurescence directe était réalisée chez 75,3% des patients et était revenue positive dans 40,6% des cas. Nous avons trouvé un dépôt prédominant de C3 isolé dans 73% des cas. Le dépôt d'IgA était corrélé au diagnostic du purpura rhumatoïde. Bien que la VLC soit considérée comme idiopathique dans 3 à 72% selon certaines séries [3, 8], une recherche des causes sous-jacentes est essentielle. Ces étiologies incluent : les médicaments, les infections, les tumeurs malignes, les connectivites et les autres maladies de systèmes [2, 3, 10]. Les infections, les médicaments et les néoplasies seraient les causes les plus fréquentes des VLC selon certains auteurs [2, 3, 10, 11]. Les maladies de système ou autres vascularites systémiques primitives sont représentées principalement par les connectivites (de 8 à 14% des cas, représentées principalement par le lupus érythémateux systémique et le syndrome de Gougerot Sjogren) [2, 3], le purpura rhumatoide (de 8,8 à 11,8%), la périartérite noueuse (de 3,2 à 6%), les vascularites urticariennes hypocomplémentémiques (de 3,2 à 5,3%), les cryoglobulinémies mixtes essentielles (1,1 à 7% des cas), la maladie de Behcet (de 2 à 6%), les maladies inflammatoires chroniques de l'intestin (3 cas rapportés par Katoprak et al. [4]). L'association de VLC avec les agents infectieux est connue depuis des décennies. Toutes les infections, qu'elles soient bactériennes, virales, fongiques ou parasitaires, sont susceptibles d'être associées à une VLC [10]. Les virus (hépatites B et C, parvovirus B19, cytomégalovirus) et les infections streptococciques sont parmi les causes les plus fréquemment retrouvées [10]. Une cause médicamenteuse est évoquée chez 8,8 à 20% des patients [3, 10] ayant une VLC. La VLC survient en moyenne trois semaines après le début du traitement médicamenteux causal avec des extrêmes de deux jours à dix ans [8]. Tai et al [2] montre que 17,2% des VLC étaient liées à une prise médicamenteuse avec une prépondérance des antibiotiques (56,3%). Chez nos patients 5,9% uniquement des VLC étaient d'origine médicamenteuse. Ce taux est considéré faible par rapport aux données de littérature. Ceci serait probablement dû aux difficultés de l'imputabilité des médicaments dans la survenue des vascularites. Les pénicillines sont les premiers médicaments responsables de la VLC [10]. Les macrolides ont également été rapportés [12]. Nous avons noté dans notre série deux cas de VLC aux pénicillines. Outre les antibiotiques, les anti-inflammatoires non stéroidiens sont aussi considérés parmi les classes de médicaments les plus incriminées des VLC [8]. Nous avons observé un cas de VLC associé à l'acide méfénamique. En dehors de ces médicaments précités, d'autres traitements ont été à l'origine des VLC telle que la Gabapentine [13]. Les vascularites paranéoplasiques constituent 30 à 40% des vascularites cutanées [14]. La VLC est considérée comme la forme la plus fréquente des vascularites cutanées paranéoplasiques. La vascularite peut être diagnostiquée simultanément avec la maladie, la précéder ou survenir au cours de son évolution. Il s'agit surtout de leucémie à trileucocytes, de syndromes myélodysplasiques et beaucoup plus rarement des lymphomes [11, 15]. Dans notre série, nous avons diagnostiqué 3 cas de VLC paranéoplasique qui étaient dues aux hémopathies malignes. Des résultats similaires aux nôtres ont été rapportés par Beylot et al qui ont recensé 12 observations de VLC et d'hémopathies, myéloïdes chez 7 patients, lymphoïdes chez les 5 autres patients [11]. La VLC a été rapportée associée également à une leucémie lymphoide chronique [16, 17], à un cancer de l'œsophage [2] ou encore à une gammapathie monoclonale.

Les VLC ont été décrites au cours des dermatoses neutrophiliques notamment le syndrome de Sweet dans 18% des cas. Dans notre série, nous avons noté une VLC associée à un syndrome de sweet chez 12,72% des patients et un seul cas de pyoderma gangrenosum. En effet, aucun cas de pyoderma gangrenosum à nos connaissances n'a été rapporté dans la littérature. La VLC est considérée comme idiopathique dans 3 à 72% selon les séries [2, 3, 11]. Dans notre série, 35, 3% des patients avaient une VLC idiopathique. L'évolution de la VLC est en général favorable. Sept pour cent des patients uniquement ont une évolution fatale. L'évolution peut se faire vers des poussées répétées pendant des mois voire des années. Une évolution aigue inférieure à 3 mois est notée chez 47 à 60% des patients selon les séries [2, 3]. Selon certains auteurs [2, 3, 8], les paramètres liés à une évolution chronique seraient : Le sexe féminin, l'âge avancé, le caractère idiopathique, la présence d'arthralgies, l'existence de lésions ulcéreuses, l'étiologie infectieuse, le facteur rhumatoïde augmenté, les anomalies du complément, et les cryoglobulinémies. Chez nos patients, deux facteurs étaient liés à une évolution aigue : l'existence d'une infection récente ou d'une prise médicamenteuse connue et deux facteurs liés à une évolution chronique notamment, les anticorps antinucléaires et la cryoglobulinémie. La décision thérapeutique doit prendre en considération l'étendue des lésions, les étiologies sous-jacentes et la sévérité de la maladie [8]. Pour les VLC médicamenteuse, l'arrêt du médicament causal peut suffire pour obtenir la guérison au bout de quelques jours [18]. Les mesures d'hygiène doivent être expliquées aux patients incluant la diminution des facteurs d'exacerbation de la vascularite tels que la station débout prolongée, l'exposition au froid, et le port de vêtements serrés, le repos en surélevant les jambes tout en gardant les membres au chaud [18]. Plusieurs traitements symptomatiques peuvent être proposés aux patients : les antalgiques, les anti-inflammatoires non stéroidiens et les antihistaminiques [1, 8]. Pour les VLC à évolution chronique ou persistante, la dapsone et/ou la colchicine peuvent être efficaces [8, 19]. L'utilisation de la colchicine dans le traitement de la VLC est bénéfique par le biais de son effet sur la diminution du chimiotactisme des polynucléaires neutrophiles, sur le blocage de l'adhésion des leucocytes et sur la stabilisation des membranes lysosymiales [19]. La colchicine à la dose de 0,6-1,8 mg/jour induit une résolution de la VLC en une à deux semaines selon plusieurs auteurs [8, 19]. Plusieurs études rétrospectives monocentriques ont démontré l'efficacité de la colchicine dans le traitement des VLC [18]. La meilleure indication de la colchicine en cas de VLC est la vascularite de sévérité modérée. C'est un traitement efficace et peu coûteux de la VLC qui peut être utilisé en monothérapie ou en combinaison [19]. Cependant sa prescription est parfois limitée en raison de ses effets secondaires gastro-intestinaux. En cas d'atteinte cutanée sévère nécrotique et/ou de manifestations systémiques, une corticothérapie par voie générale (prednisolone ou prednisone 20-60mg/j) avec dégression progressive peut contrôler la situation dans certains cas [1, 2, 20]. Pour les patients ayant des manifestations systémiques le traitement initial doit inclure des doses élevées de corticoïdes et/ou le cyclophosphamide [1]. Des immunoglobulines intraveineuses peuvent être utiles dans le traitement des VLC sévères et réfractaires chez les patients ayant une contre indication au traitement immunosuppresseur traditionnel [20].

## Conclusion

Notre étude confirme les données de la littérature concernant les caractéristiques épidémio-cliniques des VLC ainsi que le profil étiologique de cette affection. En effet, il existe une multitude d'étiologies des VLC secondaires permettant ainsi de suggérer d'adapter une stratégie dans l'enquête étiologique en insistant sur les maladies de système, les causes néoplasiques et médicamenteuses. Une identification des facteurs pronostiques est par ailleurs souhaitable afin de pouvoir rapidement moduler la prise en charge thérapeutique et agir ainsi sur le l'évolution de cette affection.

### Etat des connaissances actuelles sur le sujet

- Les étiologies des vascularites cutanées leucocytoclasiques sont très nombreuses;
- Les facteurs prédictifs de l'évolution des VLC sont peu étudiés.

### Contribution de notre étude à la connaissance

- les maladies systémiques, les infections (20%) et les dermatoses neutrophiliques sont les étiologies les plus fréquentes des vascularites cutanées leucocytoclasiques;
- Les facteurs prédictifs à l'évolution aigue des vascularites cutanées leucocytoclasiques: la présence d'une infection récente et l'étiologie;
- Les facteurs prédictifs à l'évolution chronique : la positivité des anticorps antinucléaires, et la cryoglobulinémie.
